# Towards high-bandwidth organic photodetection based on pure active layer polarization

**DOI:** 10.1038/s41598-018-33822-z

**Published:** 2018-10-18

**Authors:** Louisa Reissig, Simon Dalgleish, Kunio Awaga

**Affiliations:** 1 Department of Chemistry and IRCCS, Nagoya University, Furo-cho, Chikusa, 464-8602 Nagoya Japan; 2 Institute of Experimental Physics, Freie Universität Berlin, Arnimallee 14, 14195 Berlin, Germany; 3 Institute for Advanced Research, Nagoya University, Furo-cho, Chikusa, 464-8601 Nagoya Japan

## Abstract

Organic photodetectors offer distinct advantages over their inorganic analogues, most notably through optical transparency and flexibility, yet their figures-of-merit still lag behind those of inorganic devices, and optimization strategies generally encounter a trade-off between device responsivity and bandwidth. Here we propose a novel photodetector architecture in which an organic photoactive semiconductor layer (S) is sandwiched between two thick insulating layers (I) that separate the semiconductor from the metallic contacts (M). In this architecture a differential photocurrent response is generated purely from the polarization of the active layer under illumination. Especially for an asymmetric MISIM design, where one insulating layer is a high-*k* ionic liquid I_IL_ and the other a low-*k* polymer dielectric I_p_, the responsivity/bandwidth trade-off is broken, since the role of the I_IL_ in efficient charge separation is maintained, while the total device capacitance is reduced according to I_p_. Thus the benefits of single insulating layer differential photodetectors (MISM) using either I_IL_ or I_p_ are combined in a single device. Further improvements in device performance are also demonstrated by decreasing the series resistance of the photoactive layer through semiconductor:metal blending and by operation under strong background light.

## Introduction

The importance of insulating materials in the advancement of organic photodiodes is often overshadowed by the plethora of literature devoted to the design and processing of the light harvesting organic semiconductor component(s) that serve as the locus of light to energy conversion in such devices^1,2^. However, insulators have played a pivotal role in performance enhancement both for solar cells and for photodetectors based on a 2-terminal photodiode structure. Applied as thin interlayers at the electrode/semiconductor interface, various insulating materials have been used to form selective contacts for both holes^3,4^ and electrons^5–7^, even for the same electrode material^7,8^. Insulators have also been used as blending partners with the semiconductor materials, leading to enhanced performance and operational stability from thicker active layer films, and thereby realizing truly plastic electronic devices^9,10^. Pertinent to photodetectors, which are generally operated under strong reverse bias, thin insulating interlayers have been shown to dramatically reduce the dark current in devices^11–13^, which constitutes the dominant component to electronic noise^14,15^ and, accordingly, such devices have shown impressive values of detectivity and noise-equivalent power - both important figures of merit for photodetector technology. However, in all these examples, the insulator component should be sufficiently thin, or sufficiently percolated by the semiconductor, to allow the passage of a DC current upon illumination. An alternative use of insulator interlayers is to act as a blocking dielectric to accumulate free carriers formed in the semiconductor layer upon photoexcitation, and to generate a transient photocurrent response due to capacitive charging/discharging of the thick insulator dielectric^16^. The bipolar current response to light on/off is similar to that of a voltage differentiator, but where the driving voltage is the photovoltage generated across the semiconductor layer^17^. While unsuitable for DC applications, such as solar cells, such differential photodetectors are highly applicable to AC devices such as optical signal receivers.

Previous work on differential photodetectors has independently demonstrated device bandwidths up to 1 MHz^18^ and responsivities reaching 272 mA/W^19^, by using low- or high-*k* dielectric insulators, respectively, as the interlayer between the semiconductor and a metal electrode in a metal/insulator/semiconductor/metal (MISM) device architecture. Typically, organic polymer dielectrics have served as representative low-*k* dielectrics, while ionic liquids (ILs) have been used for enhanced responsivity due to the large capacitance of the electric double layers (EDLs) that form at the ionic liquid interfaces, as well as the associated large electric field developed at the S/I_IL_ interface that can enhance charge separation in the semiconductor following photoexcitation^17^. However, a trade-off has existed in these figures of merit due to the constraints imposed on the single dielectric layer. This trade-off has been demonstrated by Hu *et al*. for MISM devices based on solid-state dielectrics^20^, and while the ionic liquid-based devices have shown improved responsivities compared to solid-state devices, their bandwidths are significantly reduced^19^ (*c.f*. Supplemental Fig. S2). While a high-*k* dielectric can accommodate more charge at the S/I interface, the non-negligible charging time of the semiconductor layer (with its own RC time constant) is slowed accordingly by the changing electric field from the insulator layer, which is slower for high-*k* dielectrics, thus permitting only mutually opposed optimization of responsivity or bandwidth^17^.

In this article we demonstrate a strategy to break the responsivity/bandwidth trade-off in differential photodetectors, exploiting the photocurrent enhancement observed in the IL based MISM devices while maintaining the bandwidth of the low-*k* polymer devices. Recognizing the fact that charge extraction at the M/S interface is not a prerequisite for differential photocurrent generation, we explored the use of an additional polymer insulator layer (I_P_) introduced between the semiconductor layer and the electrode, yielding a device structure of MI_IL_SI_P_M. For such devices, the strong electric field at the S/I_IL_ interface is largely maintained, supporting efficient charge separation across the active layer, while the series capacitance of the device is significantly reduced according to I_P_ (since 1/C_tot_ ≈ 1/C_P_ when C_IL_ ≫ C_P_), thus the bandwidth can be expected to approach that of MISM devices based on low-*k* dielectrics. In this architecture, the photocurrent response is generated purely by the polarization of the semiconductor layer under illumination, without carrier injection from the electrode(s), thereby eliminating contact resistance effects that can additionally reduce the bandwidth and the responsivity of MISM devices. By this method, the divergent optimization strategies for MISM photodetectors converge in a platform that exploits the advantages of both insulator layer functions.

**Figure 1:**
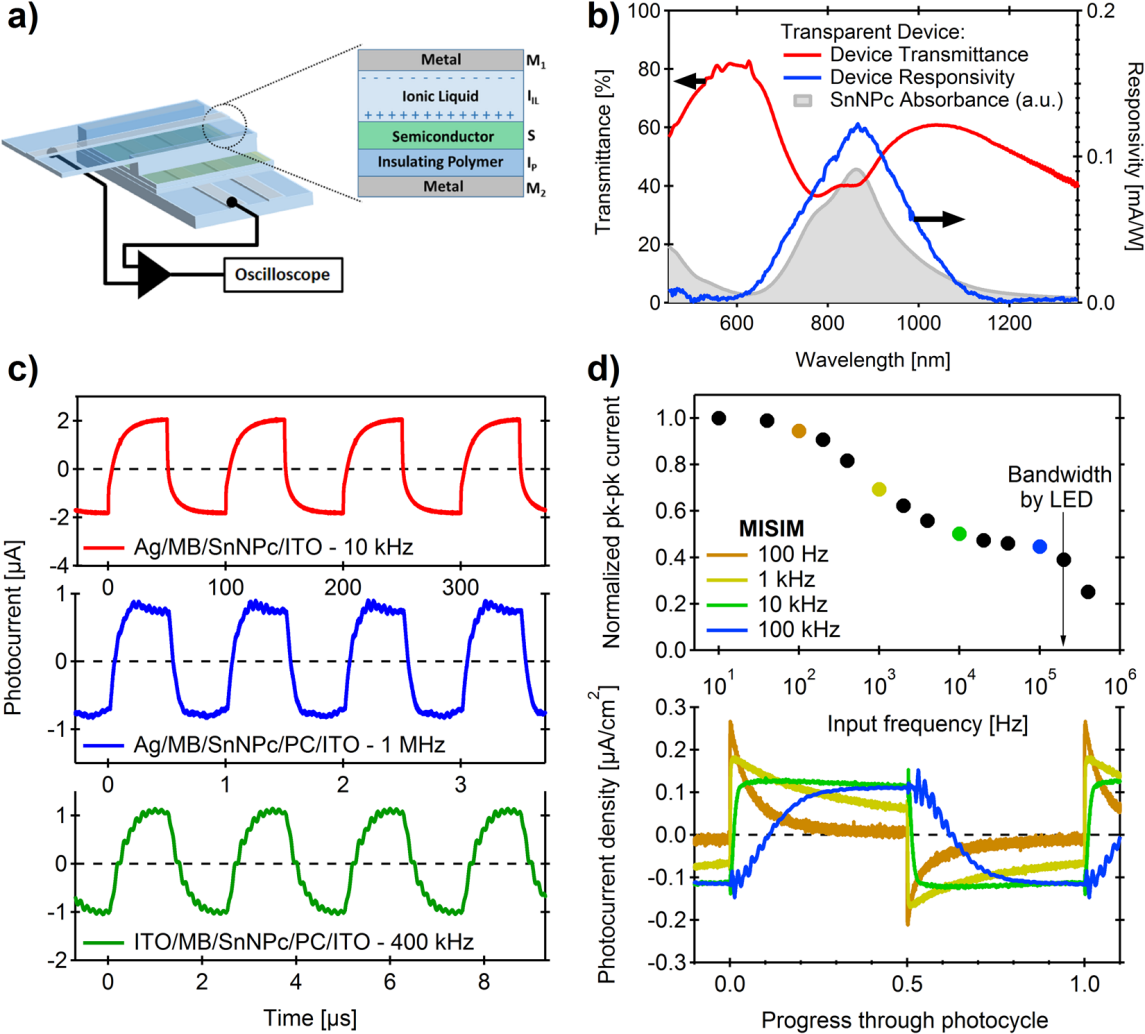
(**a**) Generalized structure of the M_1_I_IL_SI_P_M_2_ devices used in this study (M_1_I_IL_SM_2_ devices are identical, except that I_P_ is absent); (**b**) responsivity spectrum for the transparent device (M_1_ = M_2_ = ITO, I_IL_ = MB, S = 50 nm SnNPc, I_P_ = 200 nm PC), compared to the transmission spectrum through the whole device stack. The absorption spectrum of the SnNPc film is shown as the shaded area under the responsivity curve, in arbitrary units, for comparison; (**c**) photocurrent response for representative M_1_I_IL_SI_P_M_2_ devices, where M_1_ = Ag or ITO, compared to M_1_I_IL_SM_2_ (M_1_ = Ag) under pulsed laser diode illumination (50% duty cycle, *λ* = 850 nm, *P*_*MAX*_ = 18 mW at 1 MHz, 19 mW at 400 kHz and 34 mW at 10 kHz) at pulse frequencies resulting in an approximately square-wave response; (**d**) progression of photocurrent response for the M_1_I_IL_SI_P_M_2_ device, where M_1_ = ITO, under square-wave modulated LED illumination (*λ* = 850 nm, *f* = 0.1–100 kHz, *P* = 1.1 mWcm^−2^).

For the purposes of this study, Sn(II)-2,3-naphthalocyanine (SnNPc) was used as the active semiconductor component due to its strong Q-band absorption centred around 850 nm, which is ideally placed for short distance fibre-optic communication. Despite its low charge carrier mobility (*µ*^+^ ≈ 5 × 10^−5^ cm^2^V^−1^s^−1^), SnNPc has previously been shown to yield reasonable responsivities for single component semiconductor layers in IL-based MISM devices for a range of ionic liquids, such as *N*-(2-methoxyethyl)-*N*-methylpyrrolidinium tetrafluoroborate (MB) or trimethylpropylammonium bis(trifluoromethanesulfonyl)imide (TT), with sufficient stability to allow comparative studies^21^. Figure 1 illustrates the design of a typical MI_IL_SI_P_M photocell used in this study (M_1_ = Ag, I_IL_ = MB, S = 50 nm SnNPc, I_P_ = 200 nm Parylene-C (PC), M_2_ = indium tin oxide (ITO)), together with its representative photocurrent characteristics (a full description of device assembly and photocurrent measurements can be found in the Supporting Information). It should be noted that such IL-based devices can be constructed with ITO serving as both electrodes, which results in a visible-transparent photodetector with a maximum transmittance of 82% at 590 nm, and 41% at its responsivity maximum of 870 nm (for a 50 nm film of SnNPc), as shown in the transmittance spectrum and responsivity spectrum of Fig. 1b. Compared to the absorption spectrum of SnNPc (shaded area, Fig. 1b), the device transmittance shows greater attenuation for light at 780 nm due to scattering and/or interference effects through the various layers of the transparent device, which accounts for the suppressed photocurrent contribution of the blue shoulder of the Q-band absorption in the SnNPc film.

Similar to previous MISM photodetectors, the photocurrent response of the MI_IL_SI_P_M devices show a characteristic progression with frequency of light signal (under square-wave modulated LED illumination), from a transient waveform at low frequencies (where complete capacitive charging of the device can be achieved), to become square-wave over a range of frequencies until the rise-time of the photoresponse is exceeded, whereupon the signal magnitude drops sharply (Fig. 1d). The device bandwidth (BW) can be calculated from the photocurrent rise time (τ_rise_) as BW = 0.35/τ_rise_, which is shown to correspond well to the point at which the photocurrent response drops below 70% of that at square-wave^13^, and which can be further improved by using a pulsed laser for device excitation (50% duty cycle), due to its smaller spot size (which yields a reduced geometric capacitance from a smaller active device area) (Fig. 1c). The MI_IL_SI_P_M photodetectors are best compared to their MI_IL_SM analogues at square-wave where the responsivity becomes frequency independent^18^, which occurs at ~1 MHz for MI_IL_SI_p_M and ~10 kHz for MI_IL_SM. While the bandwidth for MI_IL_SI_p_M of 3.1 MHz is almost 100x larger than that of the analogous MI_IL_SM (31 kHz), its responsivity is smaller by only a factor of 1.2 (Fig. 1d). As a convenient metric, we introduce the responsivity-bandwidth product (RBP) (*c.f*. gain-bandwidth product) as a means to compare the devices. It is clear that, despite its reduced responsivity, the MI_IL_SI_P_M device improves upon MI_IL_SM in this metric due to a significant improvement in its bandwidth, yielding a RBP of 290, compared to 3.5 for the MI_IL_SM device.

**Figure 2:**
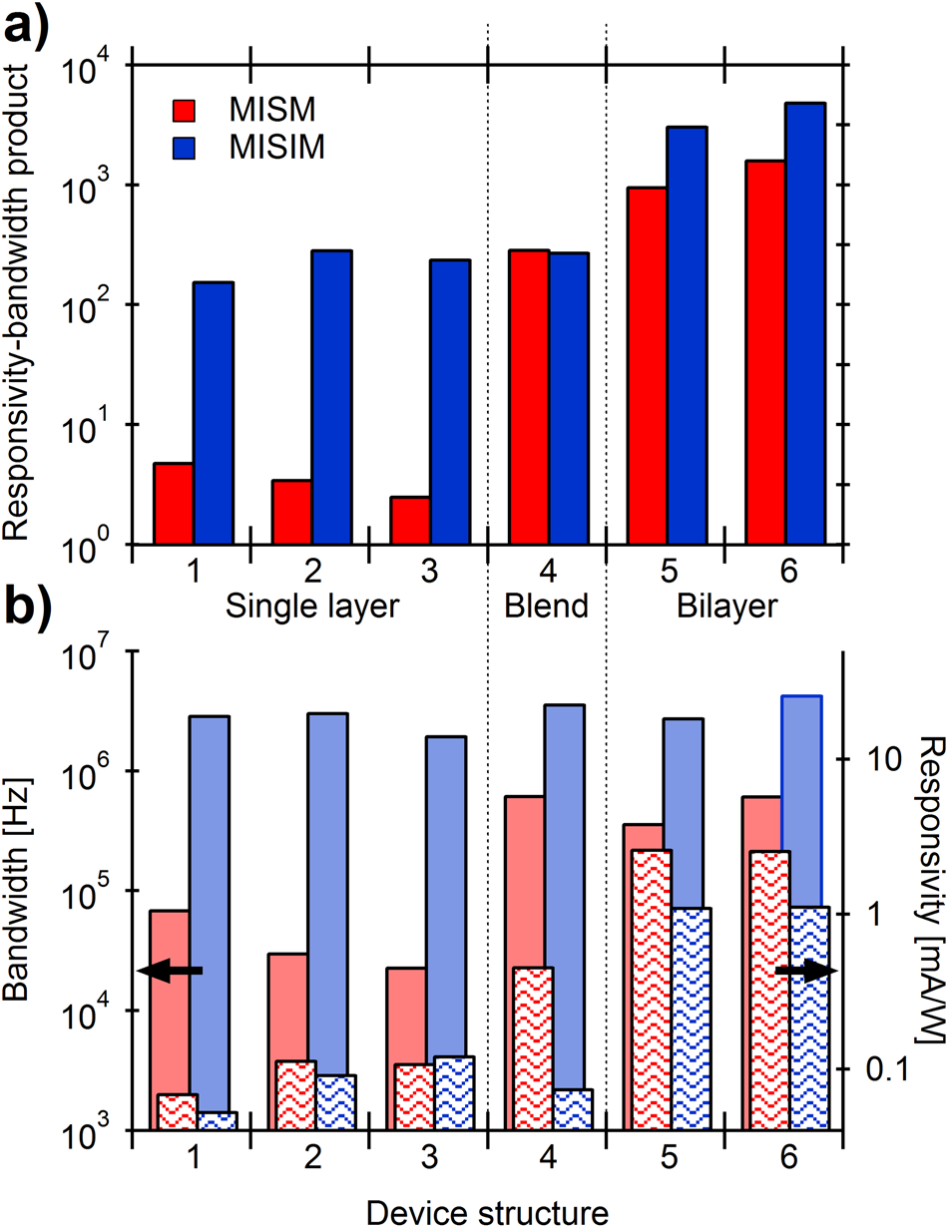
(**a**) Responsivity-bandwidth product (RBP) comparison of MI_IL_SM and MI_IL_SI_P_M devices for various semiconductor layer arrangements based on SnNPc as a single layer (Dev. 1–3), as a blend (Dev. 4) and as a bilayer (Dev. 5–6) with PTCBI. Dev 1 (see also Fig. 1): ITO/(PC)/SnNPc(50 nm)/MB/Ag; Dev 2: ITO/MB/SnNPc(50 nm)/(PC)/Ag; Dev 3: ITO/(PMMA)/SnNPc(60 nm)/TT/Ag; Dev 4: ITO/(PMMA)/SnNPc:PTCBI(2:1,60 nm)/TT/Ag; Dev 5: ITO/(PC)/SnNPc(40 nm)/PTCBI(20 nm)/MB/Ag; Dev 6: ITO/MB/SnNPc(40 nm)/PTCBI(20 nm)/(PC)/Ag; (**b**) separate values of responsivity and bandwidth for the same devices.

To demonstrate the generality of improvement in RBP of MI_IL_SI_P_M over MI_IL_SM, various device configurations were tested based on SnNPc as a single component semiconductor layer, as well as bilayer and blend films with 3,4,9,10-perylenetetracarboxylic bisbenzimidazole (PTCBI). In all cases, the electrodes (M) were either ITO or Ag, I_P_ was a 200 nm thick layer of either poly(methyl methacrylate) (PMMA) or PC, and I_IL_ was either MB or TT. Figure 2 shows the RBP comparison for MI_IL_SM and MI_IL_SI_P_M devices, together with their absolute values of responsivity and bandwidth.

In most cases, the MI_IL_SI_P_M architecture yields a significant improvement in RBP, compared to MI_IL_SM. Notably, in the case of the single layer devices (Dev. 1–3), the improvement in RBP can be up to 1–2 orders of magnitude, owing to a similar responsivity at square wave, coupled with a significantly improved device bandwidth, in comparison to the blend or bilayer devices, where only a negligible or small improvement in RBP can be seen, respectively. Here, it is reasoned that the EDL, which can provide a sufficient energetic interface for charge separation^17,21^, yields a more dominant effect in the single active layer devices (Dev. 1–3) due to the absence of any other such energetic interface, *e.g*. a heterojunction or rectifying M/S contact, as are present in the bilayer/blend devices, and the MISM devices, respectively. The function of the EDL seems preserved in MI_IL_SI_P_M devices, despite the modified potential drop across the IL with the introduction of the I_P_ (see further for discussion on the built-in potentials in MI_IL_SI_P_M). Therefore, the dominant effect of I_P_ is to reduce the series capacitance of the device, which manifests itself not just in the decay time of the photocurrent transient, but also in the photocurrent rise time^17^. An additional advantage of the MI_IL_SI_P_M structure is that the polymeric insulator layer can be used as a spacer to move the semiconductor away from the electrode to mitigate against the diminishing optical electric field close to reflective electrode contacts (optical interference effect)^22,23^. This perhaps accounts for the similar responsivity between MI_IL_SM and MI_IL_SI_P_M in Device 2 which, in turn, affords the largest improvement in RBP.

Interestingly, the MI_IL_SI_P_M architecture yields a similar device bandwidth, regardless of the semiconductor layer arrangement used. This is distinct from the MI_IL_SM devices, which show a strong dependence of bandwidth with the semiconductor layer structure employed. This suggests that the introduced I_P_ layer constitutes the smallest capacitive element in the MI_IL_SI_P_M devices, and thus dominates C_rise_. Furthermore, it should be noted that for all MISIM devices it is the more mobile charge carrier (holes for SnNPc and electrons for PTCBI) that are accumulated at the S/I_IL_ interface, regardless of the electrode arrangement used (in contrast to the MISM analogues), opening a question on the role and orientation of the built-in potentials in the MISIM devices - a question which requires further studies. This unipolar movement of charge solves an outstanding issue for the MISM photodetectors where, for certain active layer arrangements, a bipolar (double peak) response can occur during the light on and off period^21^. Such double peak behaviour has occurred in MISM devices based on single layers, bilayers and bulk heterojunction devices for various active layer materials^24,25^. While this double peak has been used by Gautam *et al*. as the basis for an elegant colour-sensitive detector^26^, this effect generally comes at the expense of device responsivity.

From Fig. 2 it can be seen that the bilayer devices show the highest values of RBP for all of the architectures tested. However, the requirement of finding a suitable heterojunction partner, which becomes harder for lower bandgap absorbers, imposes a limit on this architecture as a general strategy for near-infrared photodetection. Due to the pronounced increases in RBP for the single layer devices, we sought to explore methods to further improve the performance of MI_IL_SI_P_M based on single component semiconductor layers by decreasing the series resistance of the active layer. Based on previous work, such a strategy can be expected to yield a simultaneous improvement in the device bandwidth and responsivity^17^.

**Figure 3:**
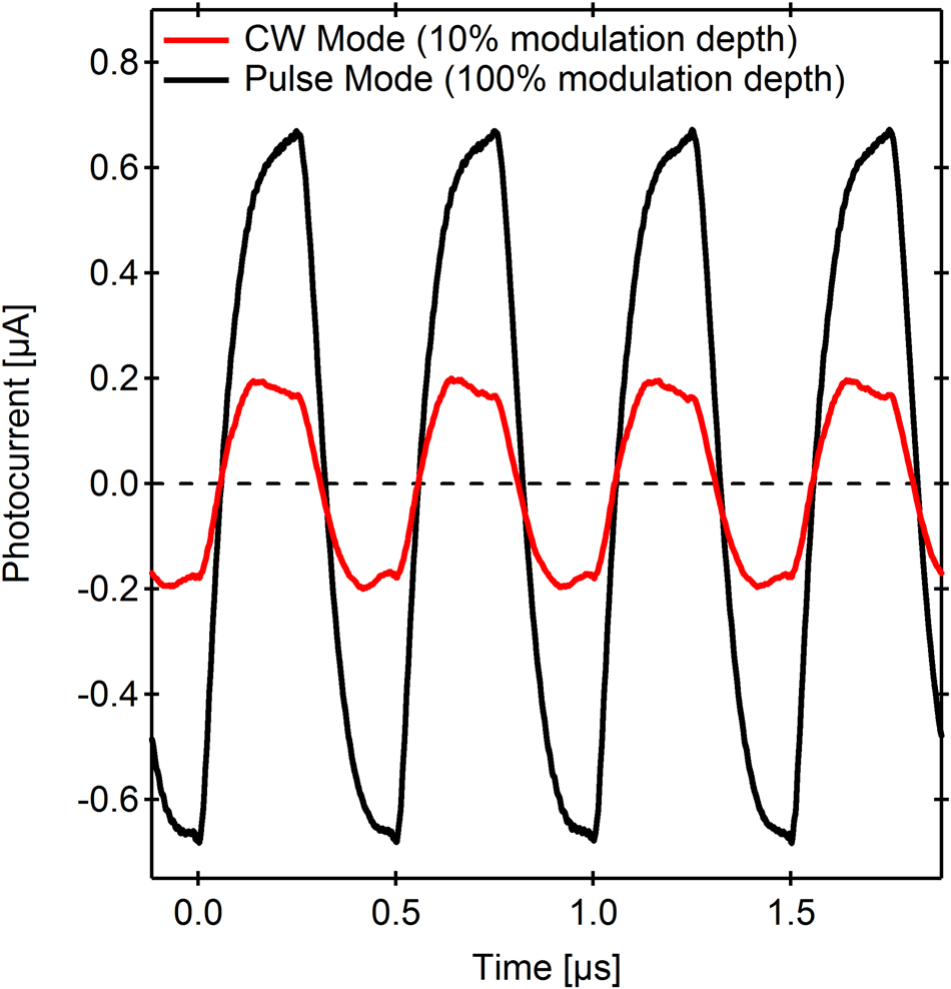
Photocurrent waveforms for Device 3, (see also Fig. 2) measured at 2 MHz using pulsed laser light illumination (50% duty cycle, *P* = 28 mW), compared to square-wave modulated CW laser light (*P* = 14 mW, *P*_*Mod*_ = 1.4 mW) at 10% modulation depth.

A key advantage of differential photodetector devices (both MISM and MISIM) is their ability to operate under strong background light conditions, since the devices are only sensitive to changes in light intensity, rather than the light intensity itself^17^. Under such conditions, a decrease in the series resistance component of the active layer could be expected due to an increased charge carrier density through pre-illumination of the semiconductor layer. As a proof of principle, the pure semiconductor device (Dev. 3 from Fig. 2) was tested under continuous wave (CW) laser illumination at 10% modulation depth (*f* = 2 MHz, P = 14 mW, P_mod_ = 1.4 mW), and the response compared to that obtained by the pulsed laser (P = 28 mW), as shown in Fig. 3. It is clear from the lack of photocurrent offset (photocurrent oscillates around zero) that the device operates as a differentiator, even at high background light intensities. Furthermore, under modulated CW illumination, the rise time falls by a factor of 1.3 to 90 ns (BW = ~3.9 MHz), while the responsivity increases 5x, thereby demonstrating that a reduced active layer resistance strongly improves device performance despite an expected loss in responsivity resulting from optical saturation. As a result, the device shows a 6.6x improvement in RBP to over 1000.

**Figure 4:**
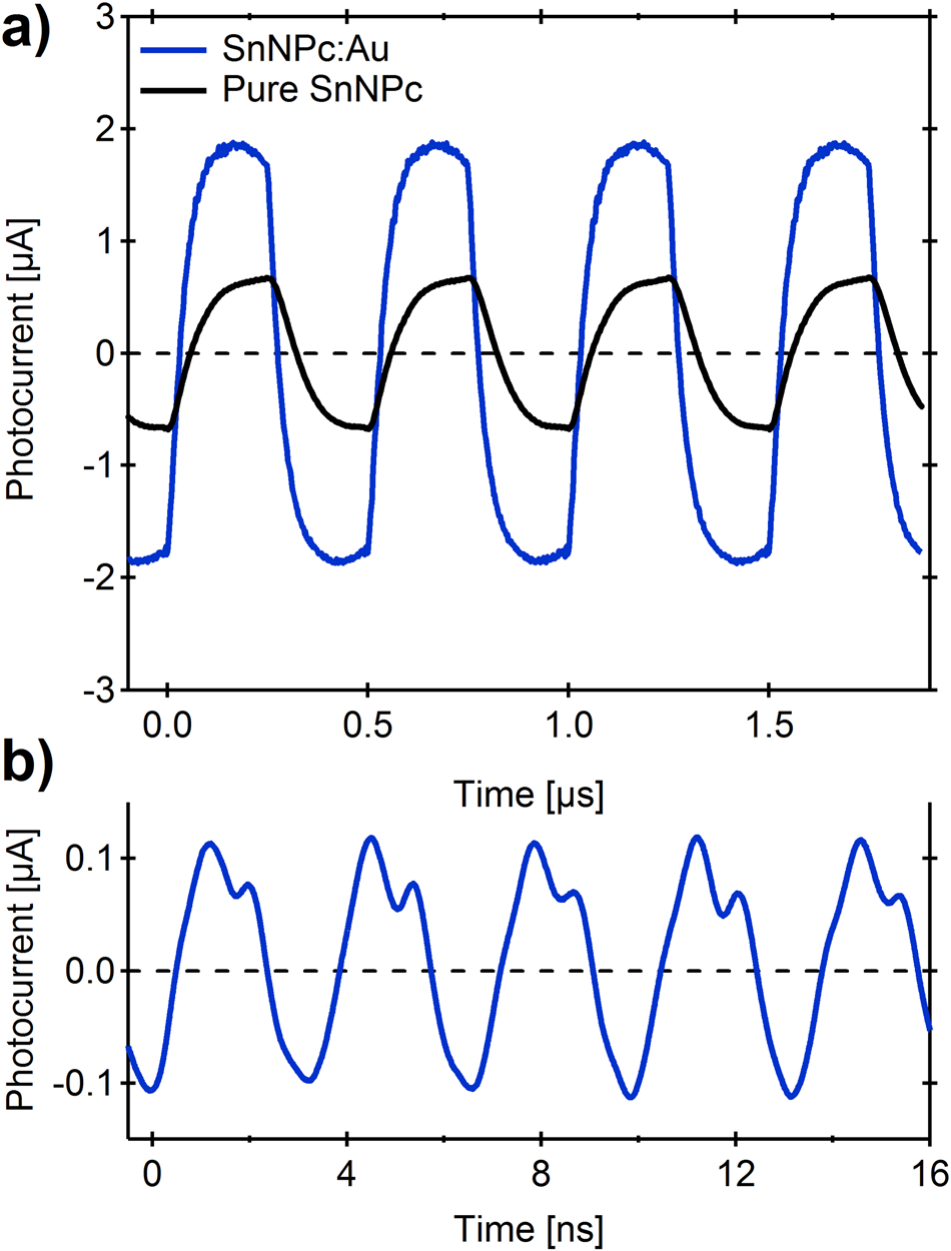
(**a**) Comparison of photocurrent waveforms for MI_IL_SI_P_M devices based on SnNPc (Dev. 3) and SnNPc:Au as the semiconductor layer, measured at 2 MHz using pulsed laser light illumination (50% duty cycle, *λ* = 850 nm, *P* = 28 mW); (**b**) photocurrent response for SnNPc:Au device measured at 300 MHz using pulsed laser light illumination (20% duty cycle, *λ* = 850 nm, *P* = 21 mW).

An alternative strategy to decrease the series resistance of the active layer, uniquely applicable to MISM or MISIM devices, was to blend directly the semiconductor with a conductive element. To effect this, a blend film of SnNPc with Au was deposited by co-evaporation, and the photoresponse compared to that of a pure SnNPc layer of identical thickness (Dev. 3 in Fig. 2) (Fig. 4a). As can be seen, the co-deposited device shows a > 2-fold improvement in photocurrent response at square-wave, while the rise time is simultaneously shortened by a factor of 1.5 to 81 ns (BW = ~4.3 MHz), yielding a RBP of 580, approaching the values obtained for the bilayer devices. It should be noted that, in contrast to the other devices in Fig. 2, where the absorption intensity at 850 nm were broadly similar, the SnNPc:Au film showed a ~2-fold reduction in absorption compared to Device 3 (see Supplementary Fig. S1). Thus using the internal quantum efficiency as a basis of comparison might be more informative in such instances. Figure 4b shows that this device is also capable of clearly resolving optical signals at frequencies up to 300 MHz (thus, far beyond its bandwidth), since the photocurrent offset is intrinsically zero, allowing signal amplification without amplifier overload. While it is premature to attribute this device improvement solely to the reduced series resistance of the device (since improved light harvesting through scattering and/or plasmonic effects might also play a role^27–29^), these results are consistent with previous work for spin-coated active layer films with incorporated carbon black^18^, and suggests metallic blending of the semiconductor layer to be a viable and general route to further improve differential photodetection. It should be noted that operating this device under CW modulation shows only negligible improvement in RBP to 620, as might be expected for an already low resistance semiconducting layer.

In this study, a general strategy to improve the device bandwidth for ionic liquid-functionalized differential photodetectors is demonstrated by adopting a MI_IL_SI_P_M architecture, where I_P_ is a low-*k* polymer dielectric. The operating mechanism for such devices is based purely on the polarization of the active layer under illumination, requiring no carrier injection from the electrodes. While a photoresponse based on pure polarization can also be achieved using two polymer dielectrics (see Supplementary Fig. S2), where electrochemistry, chemical doping and active layer dissolution are unquestionably absent, the increases in bandwidth are counterbalanced by a comparably strong decrease in responsivity, and thus the overall improvement in RBP is less pronounced than in the MI_IL_SI_p_M system. It should be noted, that by choosing the right ionic liquid for the specific active layer, a stable photocurrent response, with minimal degradation or baseline drift, can be achieved. For single layer and bilayer semiconductor systems, the improvement in bandwidth in the MI_IL_SI_p_M devices is larger than the loss in responsivity caused by a reduced total insulator capacitance, thus resulting in a net gain in responsivity-bandwidth product compared to MI_IL_SM for both these active layer arrangements. A further optimization of single component semiconductor devices is also demonstrated by reducing the series resistance of the semiconductor through operation of the device under strong background light, as well as through semiconductor:metal blending. By demonstrating device bandwidths of 4 MHz, even for a low mobility semiconductor, this study paves the way for >MHz photodetection from those organic dyes that have previously been deemed unsuitable for such purposes, but that otherwise show desirable absorption properties. Work is ongoing to assess the effect of semiconductor and insulator layer thickness on device performance, as well as the role of the built-in potentials from the outer electrode materials. In addition, improvements in both responsivity and bandwidth for MI_IL_SI_p_M devices will be targeted through the use of higher mobility and stronger chromophoric semiconductors, in an effort to tune the RC component of the photoactive semiconductor layer.

## Methods

Further information on all the fabrication and testing methods can be found in the Supplementary Information.

### Fabrication Methods

#### General Methods

All substrates were cleaned by mild bath ultrasonnication (*P* = 45 W, 0.5% Helmanix III solution, acetone, methanol, 10 mins each) and blown dry with nitrogen. Unless otherwise stated, all substrates were further subjected to UV/air plasma cleaning (Bioforce Nanosciences ProCleanerPLUS, 10 mins.) immediately prior to deposition (excepting Ag electrodes, which were freshly deposited before use).

PMMA (Mw = 350,000 – Sigma Aldrich) was spin coated from a solution of chlorobenzene (4 wt.%), and annealed on a hotplate at 160 °C for 10 mins. Parylene-C (SCS) was deposited using a Specialty Coating Systems PDS2010 deposition unit.

Organic semiconductors (SnNPc – Sigma Aldrich, PTCBI – synthesised according to Mamada *et al*.^30^) and metals (Ag, Au) were deposited by physical vapour deposition at a base pressure of <4 × 10^−4^ Pa by resistive heating of ceramic crucibles or tungsten boats, respectively. Growth rates were (independently) monitored by quartz crystal microbalance(s), and film thicknesses were validated by surface profilometry (Dektak 150).

Thin-film absorption measurements (Shimadzu UV-3100PC) were recorded on quartz substrates in transmission mode. For the device transmission measurement, a perforated plate of *ø* = 1.0 mm was used to ensure the incident light passed through the patterned ITO electrode.

#### Device Fabrication

Semiconductor films were deposited on patterned electrode (Ag or ITO) substrates (15 × 15 mm (substrate) with 2 mm wide electrode strips), either with or without a 200 nm film of polymer dielectric (PMMA or Parylene-C). The devices were assembled in a stacked architecture, where the counter electrode (either unpatterned ITO on a 10 × 20 mm glass substrate, or a 2 mm wide strip of Ag on glass of the same dimensions) was fixed on top and perpendicular to the active layer electrode with a thermally sealable 60 μm Surlyn spacer (Solaronix), as shown in Fig. 1a. The void between the electrodes was filled with ionic liquid (either *N*-(2-methoxyethyl)-*N*-methylpyrrolidinium tetrafluoroborate (MB) received as a gift from Nisshinbo Holdings Inc., or trimethylpropylammonium bis(trifluoromethanesulfonyl)imide (TT) (Kanto Chemicals)).

### Device Testing

#### Frequency dependent photocurrent measurements

For LED measurements, the LED light (*λ*_max_ = 850 nm) was directed onto a collimator via an optical fibre, resulting in a 7 mm homogeneous broad beam, which was modulated as a square-wave light signal by a function generator (Tektronix AWG2041). In all cases, the light power was adjusted to *P*_incident_ ≈ 1.1 mWcm^−2^, and the responsivity was calculated based on a device area ≈ 0.14 cm^2^ (ITO width × beam diameter).

For pulsed laser measurements, a customised laser (spot size = 2 × 1 mm, *λ*_max_ = 848 ± 10 nm, OPG-1000PL-850, Scientex) was used to generate pulsed light signals of approximately square-wave shape with a duty cycle of about 50% over a frequency range of 10 kHz to 1 GHz, controlled by a function generator (Tektronix AWG2041) (see Supplemental Fig. SX1).

For CW laser measurements, the same laser was used for generating a modulated CW waveform (using the CW trigger input). In this case, the laser power was linearly set by the laser input power (0–100%), with a constant modulation of about 10% of the maximum laser power (*i.e*. at an input power = 100%) for a laser power over 10% (see Supplemental Fig. SX2).

In all cases the generated photocurrent response was amplified using a high-speed transimpedance amplifier (Femto DHPCA100 (up to 10 MHz), or Femto HSA-Y-1-60 (2 MHz–300 MHz)), and visualized on an oscilloscope (Tektronix TDS5104B) in high resolution or average mode. The signals were displayed, so that a positive peak corresponds to the light on signal. In the case of the responsivity-bandwidth product (RBP), the responsivity was calculated using the pk-pk value of the photocurrent response for a square-wave device signal recorded at an appropriate modulation frequency, while the bandwidth was estimated from the rise time of this square-wave signal, as described in the manuscript above.

#### Wavelength dependent measurements

The devices were illuminated by a tungsten/halogen light source (Spectral Products ASBN-W 100 L) attached to a monochromator (Digikröm CM110, slits 1.2 and 0.6 mm, resolution 8–10 nm), and modulated by a chopper (NF Corp. 5584 A). The photocurrent signals were extracted using a lock-in amplifier (NF 5610B/A), pre-amplified using a low noise transimpedance amplifier (Femto, DLPCA 200) and recorded on a computer. The setup was controlled by a home-written LabVIEW program, scanning the wavelength range from 1400 to 400 nm (step ± 2 nm at 2 s/step). The responsivity spectra were determined by dividing the photocurrent spectra by the wavelength-dependent light power (see Supplementary Fig. SX3).

## Electronic supplementary material

Supplementary Information

## Data Availability

The datasets generated during and/or analysed during the current study are available from the corresponding author on reasonable request.
