# Supplementary material for: Towards high-bandwidth organic photodetection based on pure active layer polarization
Source: Sci Rep. 2018 Oct 18;8:15415. doi: 10.1038/s41598-018-33822-z (PMC6193929; doi:10.1038/s41598-018-33822-z)
Supplement: Supplementary file 1 — Supplementary Information [file 41598_2018_33822_MOESM1_ESM.pdf]

Electronic Supporting Information (ESI)

Towards high-bandwidth organic photodetection based on pure active layer polarization

Louisa Reissig[1,2]*, Simon Dalgleish[1,3], Kunio Awaga[1]*

[1] *Department of Chemistry and IRCCS, Nagoya University, Furo-cho, Chikusa, 464-8602 Nagoya, Japan*

[2] *Institute of Experimental Physics, Freie Universität Berlin, Arnimallee 14, 14195 Berlin, Germany*

[3] *Institute for Advanced Research, Nagoya University, Furo-cho, Chikusa, 464-8601 Nagoya, Japan*

CONTENTS:

1) **Materials and Methods**
2) **Supporting Data**
3) **References**

MATERIALS AND METHODS

General Methods

SnNPc was purchased from Sigma Aldrich, and used as received. PTCBI was synthesized according to Mamada *et al.*[1] and purified by repeated sublimation under vacuum (*cis* and *trans* isomers were not separated). All substrates were cleaned by mild bath ultrasonnication (P = 45 W, 0.5% Helmanix III solution, acetone, methanol, 10 mins each) and blown dry with nitrogen. Unless otherwise stated, all substrates were further subjected to UV/air plasma cleaning (Bioforce Nanosciences ProCleanerPLUS, 10 mins.) immediately prior to deposition (excepting Ag electrodes, which were freshly deposited before use).

Organic films were fabricated under physical vapour deposition (PVD) at a base pressure of $<4 \times 10^{-4}$ Pa by resistive heating of a ceramic crucible to induce sublimation. The growth rates were monitored using up to two independent quartz crystal microbalances (QCMs), calibrated as described below. In all cases, the devices fabricated for comparison between MISM and MISIM were fabricated in the same deposition run. Silver and aluminium electrodes, and gold for the semiconductor:metal blend, were deposited under PVD from a tungsten boat, under similar conditions to the organic films. Electrode patterning was achieved via a shadow mask.

PMMA (Mw = 350,000, Sigma Aldrich) was spin coated from a solution of chlorobenzene (4 wt.%), and annealed on a hotplate at 160°C for 10 mins. Parylene-C (SCS) was deposited using a Specialty Coating Systems PDS2010 deposition unit.

Film Characterization

Film thicknesses were estimated for each deposition condition (for QCM validation) by surface profilometry using a stylus-type step profiler (Dektak 150, scan speed = 4 µm/s, stylus force = 1 mg).

Thin-film absorption measurements (Shimadzu UV-3100PC) were recorded on 10 × 10 mm quartz substrates in transmission mode, with the beam profile reduced using a perforated plate (ø = 6.5 mm), and with substrates fixed normal to the incident beam. For the device

transmission measurement, a perforated plate of ø = 1.0 mm was used to ensure the incident light passed through the patterned ITO electrode.

Device Fabrication

IL-based devices – The various films for device testing were grown by single or dual source PVD (described above) on patterned electrode (Ag or ITO) substrates (15 mm x 15 mm (substrate) with 2 x 2mm electrode strips), either with or without a 200 nm film of polymer dielectric (PMMA or Parylene-C). The devices were assembled in a stacked architecture, where the counter electrode (either unpatterned ITO on a 10 × 20 mm glass substrate, or a 2 mm wide strip of Ag on glass of the same dimensions) was fixed on top and perpendicular to the active layer electrode with a thermally sealable 60 µm Surlyn spacer (Solaronix), as shown in Figure 1a of the manuscript. The devices were completed immediately prior to measurement by filling the void between the electrodes with the ionic liquid (either *N*-(2-methoxyethyl)-*N*-methylpyrrolidinium tetrafluoroborate (MB) received as a gift from Nisshinbo Holdings Inc., or trimethylpropylammonium bis(trifluoromethanesulfonyl)imide (TT) (Kanto Chemicals)). These ionic liquids were chosen based on the negligible dissolution of the active layer films in long-term contact with them, as determined by a spectroscopic method reported previously.[2]

Solid-state devices – The active layers were deposited in an identical manner to the IL-based devices, after which a 200 nm film of PVDF (Mw = 534,000, Sigma Aldrich) was deposited by PVD, and the devices completed with a 2 mm wide Al top contact deposited perpendicular to the active electrodes.

Device Testing

Four different optical configurations were used in this study:

(1) **<u>Frequency dependent photocurrent measurements</u>**:

(a) LED setup:[2,3] Light from an LED (λ_{max} = 850 nm) was directed onto a collimator via an optical fibre, resulting in a 7 mm homogeneous broad beam, which was modulated as a square-wave light signal by a function generator (Tektronix AWG2041). The light power was adjusted before each experiment by a home-built driver unit (circuit diagram available upon request), and monitored by a Si PIN diode (FID08T13TX) calibrated by an optical power meter (ADCMT 8230E). In all cases, a light power $P_{incident}$ ≈ 1.1 mWcm^{-2} was used and the responsivity was calculated based on a device area ≈ 0.14 cm^2 (ITO width × beam diameter). The measurements were made over an appropriate frequency range 1 Hz – 100 kHz.

(b) Laser setup (pulse): The devices were excited using a laser (spot size = 2 x 1 mm, λ_{max} = 848 ± 10 nm, OPG-1000PL-850, Scientex), which was customized by the company to generate pulsed light signals of approximately square-wave shape with a duty cycle of about 50% for most frequencies between 10 kHz to 1 GHz, when applying a square-wave trigger to the pulse input of the controller (trigger intensity = 1 Vpp, applied by a function generator (Tektronix AWG2041)) (see Fig. SX1). Due to the light power and duty cycle dependence on modulation frequency, the laser was calibrated using an ultra-high speed photoreceiver with integrated Si-PIN Photodiode (Femto, AS-X-S-1G4-SI), for each input power used (generally 60 or 100%), and the shape further confirmed using a conventional Si-PIN diode (without high-pass filter).

(c) Laser setup (CW): The same laser was used for generating a modulated CW waveform when the CW trigger input was connected to the function generator (trigger intensity = 0.5 V). In this case, the laser power was linearly set by the laser input power (0-100%), with a constant modulation of about 10% of the maximum laser power (*i.e.* at an input power = 100%) for a laser power over 10% (Fig. SX2), and showing a similar dependence on frequency as for the pulsed laser. Also for the CW laser, the laser power and modulation depth was calibrated using the integrated Si-PIN Photodiode and the conventional Si-PIN diode mentioned above.

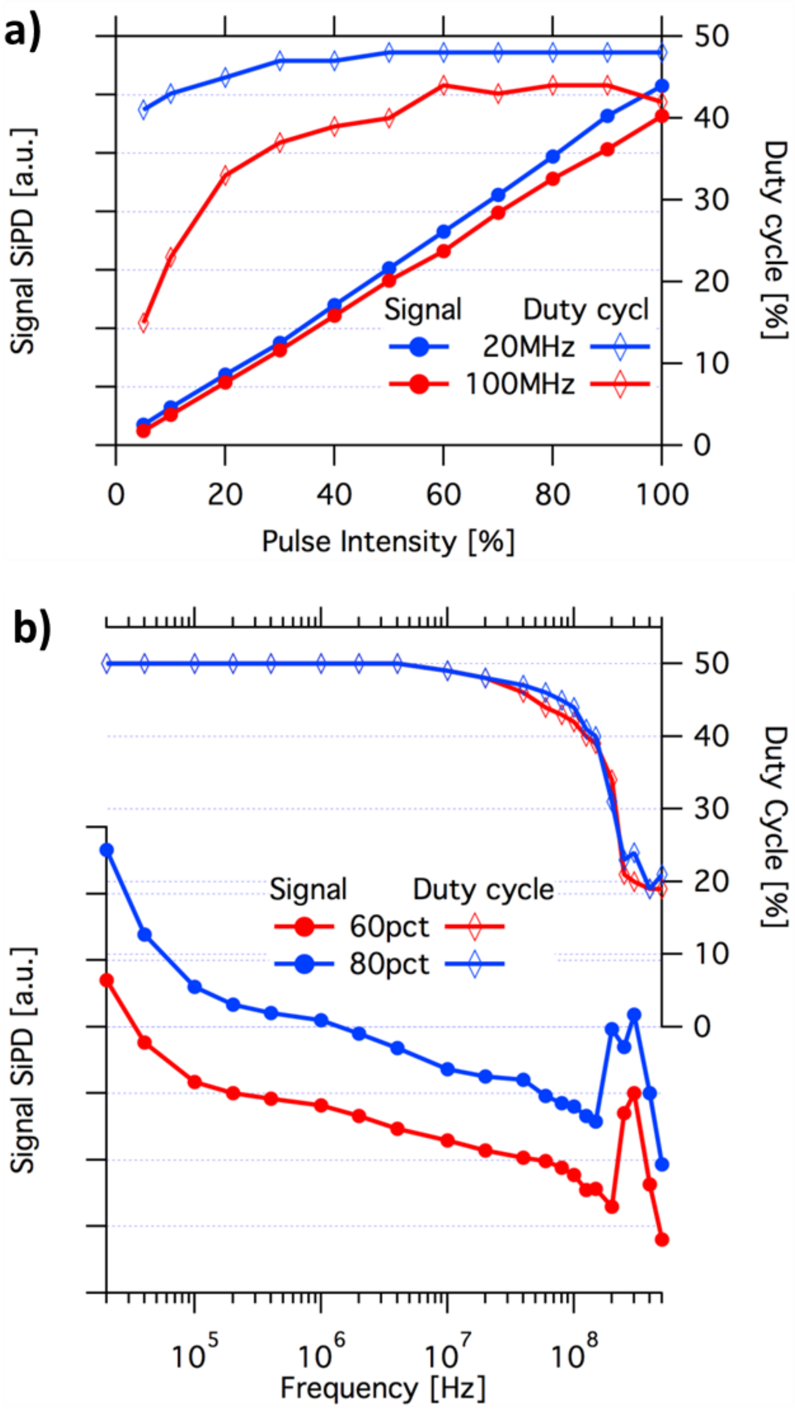

Fig. SX1: Representative characteristics of the custom modified pulsed laser: (a) dependence of light pulse power and duty cycle on set pulse intensity; (b) dependence of light pulse power and its duty cycle on modulation frequency.

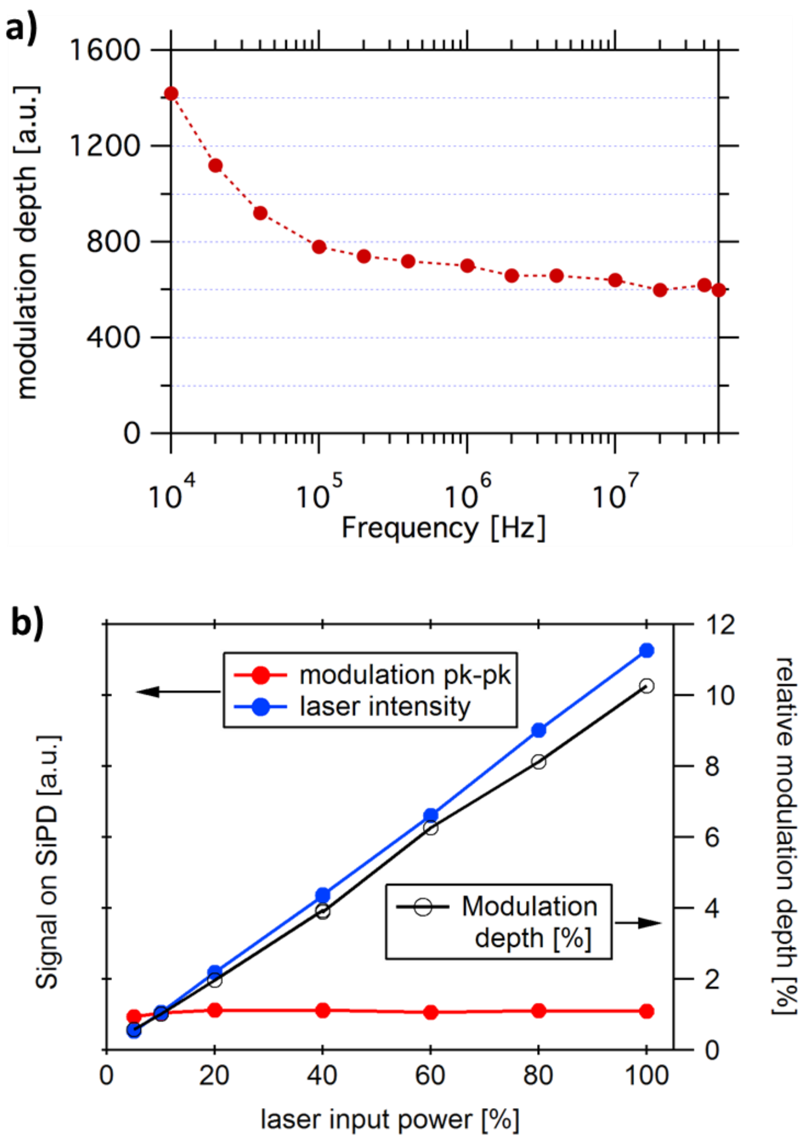

Fig. SX2: Representative characteristics of the modulated CW laser: (a) dependence of laser light power and modulation depth on set pulse intensity; (b) dependence of modulation depth (laser light power) on frequency.

In all cases the generated photocurrent response was amplified using a high-speed transimpedance amplifier (Femto DHPCA100 (up to 10 MHz), and Femto HSA-Y-1-60 (2 MHz – 300 MHz) if the bandwidth of the device or the frequency of the measurement exceeded the bandwidth of the DHPCA amplifier), and visualized on an oscilloscope (Tektronix TDS5104B) in high resolution or average mode. The stability (within 10%) within the experimental timespan was confirmed, unless otherwise mentioned. In all cases the signals were displayed, so that a positive peak corresponds to the light on

signal. It should be noted that all MISIM devices showed the accumulation of the fast charge carrier (holes for SnNPc and electrons for PTCBI) at the ionic liquid interface, while a mixture was reported for their MISM analogues, depending on active layer design and built-in potentials, as previously described.[3,4] In the case of the reponsivity-bandwidth product (RBP), the responsivity was calculated using the pk-pk value of the photocurrent response for a square-wave device signal recorded at an appropriate modulation frequency, while the bandwidth was estimated from the rise time of this square-wave signal as described in the manuscript. It should be noted that while the values between devices fabricated in different batches could vary, a clear trend in RBP enhancement was largely consistent between batches.

(2) <u>Wavelength dependent measurements (Action spectrum)</u>:[3,4]

The devices were illuminated by a tungsten/halogen light source (Spectral Products ASBN-W 100 L) attached to a monochromator (Digikröm CM110, slits 1.2 and 0.6 mm, resolution 8 – 10 nm), and modulated by a chopper (NF Corp. 5584A). The photocurrent signals were extracted using a lock-in amplifier (NF 5610B/A), pre-amplified using a low noise transimpedance amplifier (Femto, DLPCA 200) and recorded on a computer. The setup was controlled by a home-written LabVIEW program, scanning the wavelength range from 1400 to 400 nm (step 2 nm at 2s/step) in both directions. The data were analysed by averaging between the forth and back scan (which showed in general high similarity, confirming the stability of the devices), and by dividing by the wavelength-dependent light power to obtain the responsivity spectra (Figure SX3). Despite the variable light power over the wavelength range studied, it should be noted that, at such optical powers, the response of the measured devices was highly linear.

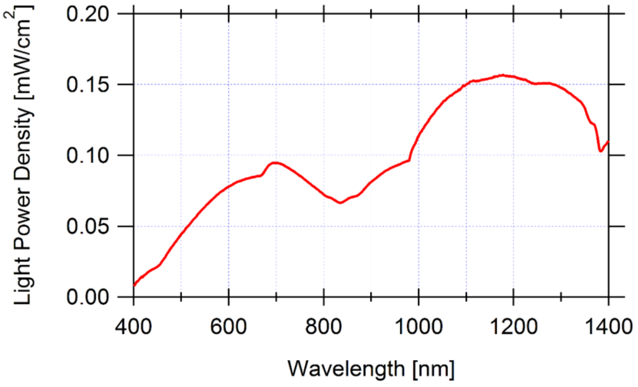

Fig. SX3: Light power spectrum used for the action spectrum measurement reported in Fig. 1b.

SUPPORTING DATA

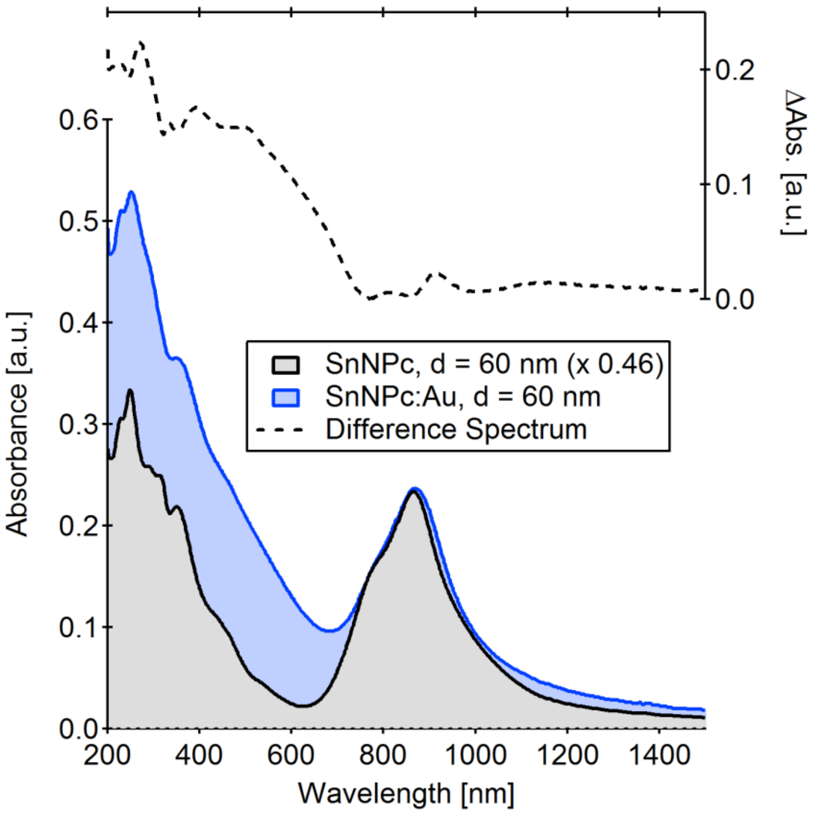

Figure S1: Thin film absorption spectra recorded on quartz substrates for a 60 nm film of SnNPc, compared to a 60 nm film of SnNPc:Au, as fabricated for the devices depicted in Figure 4 of the manuscript.

The nature of the semiconductor:metal deposition was such that some cross-contamination of the QCM monitors could not be avoided. Therefore, to verify the approximate composition of the film, absorption spectroscopy was performed for films deposited on quartz substrates (Figure S1), together with thickness measurements by profilometry. By comparison with a 60 nm film of pure SnNPc (Device 3), the NIR peak was about half as intense, suggesting an approximately 50 vol.% content of SnNPc in the semiconductor:metal blend film. The spectrum also shows a pronounced baseline curvature, typical of Raleigh scattering of small particles. From the difference spectra, no defined peak is observed, beyond the underlying undulations of the SnNPc spectrum, suggesting that the gold particles are unstructured and very small. This was further confirmed by the very similar surface profiles for both films, as measured by AFM (not shown).

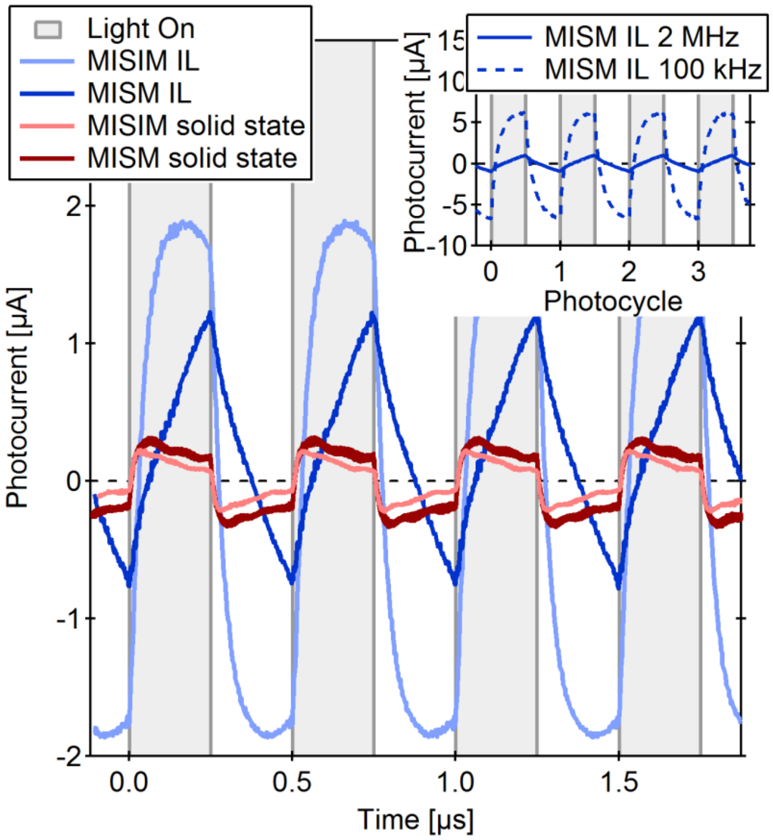

Figure S2: Comparison of the waveforms for the IL-based SnNPc:Au devices with device structures $M_1I_{IL}SI_PM_2$ and $M_1I_{IL}SM_2$ (I_{IL} = TT, I_P = PMMA, M_1 = Ag, M_2 = ITO) and the solid-state devices with device structures $M_1I_{P1}SI_{P2}M_2$ and $M_1I_{P1}SM_2$ (I_{P1} = PVDF, I_P = PMMA, M_1 = Al, M_2 = ITO). The devices are measured at 2 MHz using pulsed laser light (50% duty cycle, P = 28 mW); (inset: frequency normalized waveforms for the $M_1I_{IL}SM_2$ device at 100 kHz (square-wave) (light P = 32 mW), compared to 2 MHz (beyond bandwidth)).

In order to confirm that the photoresponse based on active layer polarization was a generalized mechanism for the MISIM architecture, and not a function of the ionic liquid (through *e.g.* electrochemical processes), several solid-state devices were fabricated and tested where I_{IL} was replaced by a relatively high-*k* solid-state dielectric, namely PVDF (in such cases the Ag counter electrode was replace with Al to avoid pin-holing of the organic films during electrode deposition). Figure S2 shows the photocurrent response for SnNPc:Au devices fabricated in the same deposition batch in both solid-state and IL-based MISM and MISIM device architectures. It is clear that a photocurrent response is also generated by the solid-state MISIM device. While the responsivity of the solid-state MISIM device is slightly smaller than for the MISM device, the bandwidth is also slightly faster. The net result is a very similar value of RBP for both devices (180 for MISM and 196 for MISIM). This contrasts

with the case of the IL-based devices, where a dramatic improvement in bandwidth of ~25x is seen for the MISIM device, while the responsivity is reduced by only a factor of 3, yielding a large improvement in RBP for the MISIM device of 580, compared to 72 for the MISM device.

Figure S2 also illustrates the existence of the responsivity/bandwidth trade-off in MISM devices. As can be seen, the photocurrent response for the ionic liquid MISM device is about 18x larger, compared to the PVDF MISM, yet the bandwidth is about 45x slower. For the MISIM device (IL/PMMA), the bandwidth is largely maintained (<1.8x slower), compared to the PVDF MISM, while the responsivity is about 6x larger.

REFERENCES

1. Mamada, M., Pérez-Bolívar, C. & Anzenbacher Jr., P. Green synthesis of polycyclic benzimidazole derivatives and organic semiconductors. *Organic Letters* **13**, 4882-4885 (2011).
2. Dalgleish, S., Reissig, L., Hu, L., Matsushita, M. M., Sudo, Y. & Awaga, K. Factors affecting the stability and performance of ionic liquid-based planar transient photodetectors. *Langmuir* **31**, 5235-5243 (2015).
3. Reissig, L., Mori, K., Treadwell, R., Dalgleish, S. & Awaga, K. Factors affecting the polarity and magnitude of photoresponse of transient photodetectors. *Physical Chemistry Chemical Physics* **18**, 6821-6830 (2016).
4. Dalgleish, S. *et al*. Controlling the crystallinity and crystalline orientation of ‘‘shuttlecock’’ naphthalocyanine films for near-infrared optoelectronic applications. *Journal of Materials Chemistry C* **6**, 1959-1970 (2018).
